# Does Nutri-Score Reliably Identify High-Sugar Foods Marketed to Children? A Cross-Sectional Study with Implications for Dental Caries Prevention

**DOI:** 10.3390/nu18142401

**Published:** 2026-07-22

**Authors:** Laura Marqués-Martínez, Carlota Rosa Pérez-Dallal, Juan Ignacio Aura-Tormos, Carla Borrell-García, Paula Boo-Gordillo, María Carmona-Santamaría, Clara Guinot-Barona, Esther García-Miralles

**Affiliations:** 1 Dentistry Department, Medicine and Health Science Faculty, Catholic University of Valencia, 46001 Valencia, Spain; laura.marques@ucv.es (L.M.-M.); carlota.perez@ucv.es (C.R.P.-D.); carla.borrell@ucv.es (C.B.-G.); paula.boo@ucv.es (P.B.-G.); maria.carmona@ucv.es (M.C.-S.); 2 Stomatology Department, University of Valencia, 46001 Valencia, Spain; juan.aura@uv.es (J.I.A.-T.); m.esther.garcia@uv.es (E.G.-M.)

**Keywords:** Nutri-Score, front-of-pack labelling, sugar content, child-targeted foods, dental caries, cariogenic potential, paediatric oral health, food labelling policy

## Abstract

**Background/Objectives**: Front-of-pack labelling systems such as Nutri-Score are promoted as public health tools to guide consumers towards healthier food choices. However, their capacity to accurately signal sugar content in child-targeted foods—a key determinant of dental caries risk—remains poorly characterised. This study aimed to evaluate whether Nutri-Score category reliably reflects sugar content in pre-packaged foods marketed to children, and to discuss the implications for paediatric oral health. **Methods**: A cross-sectional observational study analysed the nutritional labels of 100 pre-packaged foods directed at the paediatric population, all displaying a Nutri-Score label, selected from three major supermarket chains in Valencia, Spain. Products were grouped into eight predefined food categories. Sugar content (g/100 g or 100 mL), Nutri-Score category (A–E), and ordinal position of sugar in the ingredients list were recorded. Global association between Nutri-Score grade and sugar content was evaluated using Spearman’s rank correlation coefficient; category-level analyses used the Kruskal–Wallis or Mann–Whitney U test as appropriate. **Results**: A moderate statistically significant positive correlation was found between Nutri-Score grade and sugar content across all 100 products (ρ = 0.515, *p* < 0.001). In the exploratory category-level analyses, suggestive differences were observed in biscuits (H = 8.72, *p* = 0.033), milks and milk drinks (H = 9.02, *p* = 0.029), and desserts (H = 8.31, *p* = 0.016); none of these *p*-values survived Bonferroni correction for multiple comparisons (adjusted α = 0.006). Notably, some products rated A and B contained high sugar levels: one A-rated breakfast cereal reached 22.4 g/100 g, and the highest B-rated product (a flavoured milk drink) contained 22.1 g/100 g. No significant association was detected in cereals, breads, dairy products, juices, or frozen foods. **Conclusions**: Nutri-Score demonstrated limited discriminatory ability to identify high-sugar child-targeted foods consistently across food categories. These findings support recommending that paediatric dental practitioners advise caregivers to evaluate sugar content and ingredients lists beyond front-of-pack grading. Further regulatory refinement of the algorithm to specifically weight added sugar exposure in child-targeted products may be warranted.

## 1. Introduction

Dental caries remains the most prevalent chronic disease in the paediatric population worldwide, with sugar consumption constituting its primary modifiable risk factor. The World Health Organization (WHO) recommends limiting free sugar intake to less than 10% of total energy—and ideally below 5%—to reduce caries incidence in both children and adults [1].

Despite this guidance, sugar consumption in European children substantially exceeds these thresholds, partly driven by the widespread availability and aggressive marketing of ultra-processed pre-packaged foods to the paediatric population [2,3].

Front-of-pack labelling (FOPL) systems have been introduced across several European countries to help consumers make informed dietary choices. Among these, the Nutri-Score—a five-level colour and letter grading system (A to E)—has been adopted in Spain, France, Germany, Belgium, and other nations. The scale ranges from grade A (dark green), denoting the most favourable overall nutritional quality, to grade E (red), denoting the least favourable, with grades B, C and D (light green, yellow and orange) representing intermediate quality. Its algorithm assigns scores based on a composite of unfavourable nutrients (energy, saturated fat, sugars, sodium) offset by favourable components (fibre, protein, fruit and vegetable content), producing a global nutritional rating per 100 g or 100 mL [4,5].

A critical limitation of this compensatory approach is that a product high in sugar may nonetheless receive a favourable grade if other nutritional attributes are deemed adequate. This is particularly concerning for child-targeted foods, where high sugar content—especially added sugars—constitutes a direct cariogenic risk regardless of the overall nutritional profile. The European Society for Paediatric Gastroenterology, Hepatology and Nutrition (ESPGHAN) recommends avoiding free sugar exposure before age two, and limiting the frequency of sugar-containing food intake at all ages [3].

**Figure 1:**
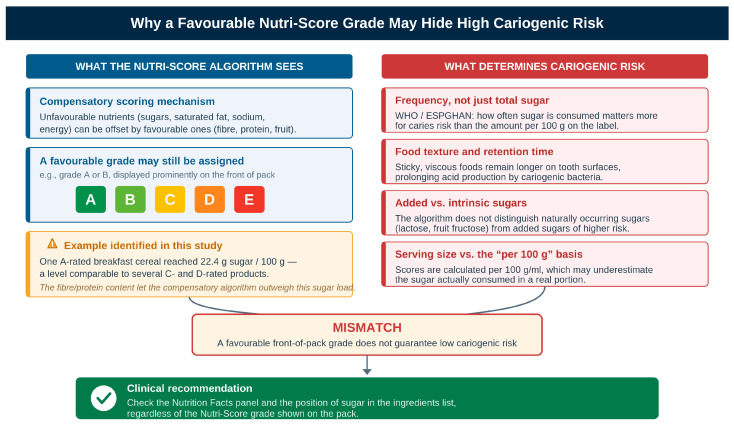
Conceptual framework illustrating how favourable Nutri-Score grades may coexist with high cariogenic potential despite favourable front-of-pack labelling.

Previous studies have identified discrepancies between Nutri-Score grades and sugar content in specific food categories. However, few have focused specifically on child-targeted foods with systematic evaluation of the implications for dental caries risk. The present study addresses this gap by conducting a cross-sectional analysis of 100 pre-packaged child-targeted foods selected from three major supermarket chains in Valencia, Spain (Figure 1) [6,7].

## 2. Materials and Methods

### 2.1. Study Design and Sampling

This was a cross-sectional, descriptive, and observational study based on the analysis of nutritional labels of pre-packaged foods marketed to the paediatric population. Data collection was conducted in two phases (November 2025 and February–March 2026) in three large retail chains (Consum, Carrefour, El Corte Inglés) in Valencia, Spain. Two data-collection phases were required to reach the predefined sample size across all eight food categories. During the second phase, products were checked for reformulation by comparing nutritional information and ingredient lists with records from the first phase; reformulated products were excluded and replaced. It should be noted that data collection took place during the transitional period following the January 2024 implementation of the updated Nutri-Score algorithm for solid foods. Because the first phase of data collection (November 2025) occurred during this official transition period, some products purchased during that phase may have displayed Nutri-Score labels calculated using the previous algorithm. Products collected during the second phase (February–March 2026) would be expected to display labels compliant with the updated algorithm, although stock turnover and individual labelling compliance could not be verified [8].

A total of 100 products were selected, all displaying a visible Nutri-Score label on packaging. Products were considered child-targeted if they met at least one of the following operational criteria: (i) presence of child-directed marketing elements on packaging (cartoon characters, bright colours, children’s promotions); (ii) belonging to food categories predominantly consumed by children (breakfast cereals, biscuits, dairy-based drinks, etc.); or (iii) explicit manufacturer declaration of child orientation. Inclusion additionally required complete nutritional information per 100 g or 100 mL. Products reformulated between collection phases were excluded. Eligible products were consecutively selected until the predefined sample size of 100 was reached. Classification of products as child-targeted was performed by a single evaluator applying the predefined operational criteria described above. The criteria were designed to be highly objective (e.g., physical presence of cartoon characters or child-directed promotional elements on the packaging, or explicit manufacturer labelling of child orientation), thereby minimising subjective interpretation. Formal inter-rater reliability was not assessed, which represents a limitation.

### 2.2. Food Categories

Products were classified into eight predefined food categories: breakfast cereals (*n* = 14), biscuits (*n* = 11), breads (*n* = 13), milks and milk drinks (*n* = 15), dairy products (*n* = 12), juices (*n* = 7), desserts (*n* = 18), and frozen foods (*n* = 10). Categories were defined according to the product type as labelled by the manufacturer, following the food groupings used in Spanish dietary guidance. The dairy product category encompassed nutritionally heterogeneous products (e.g., plain cheeses and sweetened soluble cocoa preparations); this internal heterogeneity is acknowledged as a limitation of the category-level analysis and is discussed in Section 4.2.

The frozen food category included child-targeted savoury frozen products such as mini pizzas, breaded chicken products (e.g., nuggets), breaded fish products (e.g., fish fingers), and potato-based products (e.g., shaped potato snacks).

### 2.3. Variables

Three variables were recorded per product: (1) total sugar content in g/100 g or g/100 mL, from mandatory nutritional labelling; (2) Nutri-Score category (A, B, C, D, or E) as displayed on the front of the pack; and (3) ordinal position of sugar in the ingredients list (i.e., the rank of the first sugar-related term among all listed ingredients), as an indirect indicator of the relative proportion of sugar in the formulation.

### 2.4. Statistical Analysis

Non-parametric tests were applied given the ordinal nature of Nutri-Score and the non-normal distribution of sugar data. Global association between Nutri-Score (treated as ordinal) and sugar content was evaluated using Spearman’s rank correlation coefficient across all 100 products. For category-level analyses, the Kruskal–Wallis test was used when three or more Nutri-Score grades were represented within a category; the Mann–Whitney U test was applied where only two grades were present. Statistical significance was set at *p* < 0.05. No adjustment for multiple comparisons was performed, as category-level analyses were considered exploratory; applying a Bonferroni correction (α = 0.006 for eight tests) rendered none of the category-level *p*-values statistically significant, further reinforcing their exploratory nature.

## 3. Results

### 3.1. Sample Description

Table 1 summarises the distribution of products by food category and Nutri-Score grade. All 100 products carried a Nutri-Score label. Grade D was most prevalent (*n* = 28), followed by grade C (*n* = 26). Median sugar content by Nutri-Score grade across the full sample is presented in Table 2.

The frozen food category comprised pizzas or mini pizzas (*n* = 3); breaded chicken products, including nuggets and chicken fingers (*n* = 3); breaded fish products, such as fish fingers (*n* = 2); and potato-based products, including shaped potato snacks (*n* = 2). Frozen desserts and ice creams were classified within the desserts category; therefore, the frozen food category contained only savoury products.

### 3.2. Global Association Between Nutri-Score and Sugar Content

**Figure 2:**
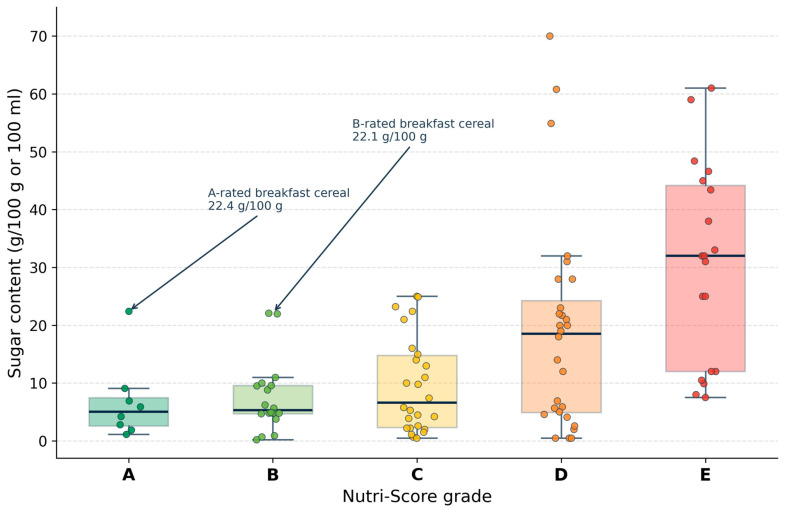
Distribution of sugar content (g/100 g or 100 mL) by Nutri-Score grade. Median sugar content increases from grade A to E, but with substantial within-grade overlap.

A statistically significant positive correlation was observed between Nutri-Score grade and sugar content across the full sample of 100 products (Spearman’s ρ = 0.515, *p* < 0.001). This indicates that products with less favourable Nutri-Score classifications generally contained higher amounts of sugar. However, substantial within-grade variability was observed, with some A- and B-rated products presenting high sugar contents (Table 2, Figure 2).

### 3.3. Category-Level Analysis

Category-level analyses showed heterogeneous patterns across food groups. In breakfast cereals, differences in sugar content across Nutri-Score grades did not reach statistical significance (Kruskal–Wallis H = 6.70, *p* = 0.082), despite a marked descriptive contrast between grades. Median sugar content was 4.2 g/100 g for grade A, compared with 22.0 g/100 g for grade B, 23.2 g/100 g for grade C and 21.7 g/100 g for grade D. Importantly, one A-rated breakfast cereal contained 22.4 g/100 g of sugar, a value comparable to those observed among C- and D-rated products.

In biscuits, an uncorrected association was observed (H = 8.72, *p* = 0.033), although this did not remain significant after Bonferroni correction. This category showed the clearest descriptive gradient, with median sugar contents of 20.0 g/100 g in grade D and 35.0 g/100 g in grade E, compared with 2.8 g/100 g in the single A-rated product. No significant association was found for breads (H = 1.98, *p* = 0.576), where sugar content remained generally low across grades, ranging from 0.5 to 13.0 g/100 g, without a discernible Nutri-Score-related gradient.

Milks and milk drinks also showed an uncorrected association between Nutri-Score grade and sugar content (H = 9.02, *p* = 0.029), but this finding should be interpreted cautiously because it did not survive correction for multiple comparisons. The pattern appeared to be largely driven by condensed milk products with very high sugar concentrations. In dairy products, no statistically significant association was detected (*p* > 0.05), and substantial within-grade heterogeneity was observed; for example, grade D included products ranging from 0.5 g/100 g in cheeses to 70.0 g/100 g in soluble cocoa products.

No significant association was observed in juices (Mann–Whitney U = 7.00, *p* = 0.857). In this category, all products contained intrinsic fructose, which appeared first in the ingredients list irrespective of Nutri-Score grade. Desserts showed an uncorrected association (H = 8.31, *p* = 0.016), with sugar content increasing descriptively across grades C, D and E; however, as with biscuits and milk-based drinks, this result did not remain significant after correction for multiple testing. Sugar values in this category ranged from 5.8 to 59.0 g/100 g. Finally, frozen foods showed no significant association between Nutri-Score grade and sugar content (H = 0.79, *p* = 0.673), with sugar levels remaining low throughout the category, with a median below 3 g/100 g.

### 3.4. Sugar Position in the Ingredients List

The ordinal position of sugar in the ingredients list showed a general trend consistent with the sugar content gradient observed in Table 2 and Table 3. Overall, 66 of 100 products (66.0%) listed a sugar-related ingredient within the first three positions. The proportion increased progressively from 37.5% in grade A products to 78.9% in grade E products (Table 4). In grade A, no product listed sugar as the first ingredient, whereas 42.1% of grade E products did so. Fourteen products (distributed across all grades) had no sugar-related term among the first listed ingredients; these were predominantly breads and frozen foods with low overall sugar content. No formal statistical comparison was performed for this variable, which was analysed descriptively.

## 4. Discussion

Overall, Nutri-Score showed a moderate positive correlation with sugar content at the aggregate level. Nevertheless, suggestive category-specific exceptions were identified, particularly among child-targeted cereals and other products receiving favourable Nutri-Score ratings despite containing high amounts of sugar; these should be interpreted with caution given the exploratory nature of the category-level analyses. Therefore, the main limitation of Nutri-Score in this context is not the absence of a global sugar gradient, but its inconsistent ability to flag individual high-sugar products within specific food categories.

These findings are explained by the algorithm’s compensatory design: favourable nutritional components—protein, dietary fibre, and fruit or vegetable content—can offset unfavourable ones, including sugars. This finding was clearly illustrated in the present study, where one breakfast cereal classified as Nutri-Score A contained 22.4 g of sugar per 100 g, a value comparable to several products classified as Nutri-Score C and D.

Consequently, a cereal enriched with fibre and protein may receive grade A despite containing over 20 g of sugar per 100 g. This mechanism has been described previously but our data demonstrate its specific relevance in the paediatric context, where sugar-driven caries risk operates independently of overall nutritional quality [6,7].

The position of sugar in the ingredients list behaved as expected under European labelling regulations, which require ingredients to be listed in descending order by weight (Regulation (EU) No 1169/2011); accordingly, this variable was closely linked to total sugar content and was not interpreted as an independent finding of the study. Its practical relevance lies elsewhere: unlike the mandatory nutrition declaration, which reports only total sugars, the ingredients list allows for identification of the type of sugar present—added sugars such as sucrose, glucose syrup or dextrose versus intrinsic sugars deriving from fruit or milk ingredients—information directly relevant to cariogenic risk that is conveyed neither by the Nutri-Score grade nor by the nutrition facts panel.

From a paediatric dental perspective, the implications are significant. Dental caries remains the most prevalent chronic disease in children worldwide, and its primary dietary driver is exposure to fermentable carbohydrates, particularly free and added sugars. Both the WHO and ESPGHAN emphasise that it is the frequency—not merely the quantity—of sugar exposure that determines cariogenic risk. These findings indicate that products with these characteristics may contribute to higher cariogenic exposure if consumed frequently, yet this risk is not signalled by the Nutri-Score grade [1,3,9].

Additional factors compound this limitation. First, Nutri-Score does not account for food texture and physical consistency; sticky or viscous foods may prolong substrate retention on dental surfaces, extending the window for acid production by cariogenic bacteria. Second, the algorithm does not distinguish between intrinsic sugars (e.g., lactose, fructose in whole fruit) and added sugars (sucrose, glucose syrups), despite evidence that the latter carry greater cariogenic and metabolic risk. Third, the system is calibrated per 100 g regardless of serving size, which may underestimate actual sugar exposure in real-world consumption contexts [6,10,11].

Our results are consistent with the previous literature. Robert et al. analysed 161 children’s breakfast cereals in France, Belgium, and Luxembourg and found that even A-rated products frequently exceeded 20 g of added sugar per 100 g. Pellegrino et al. reported that 58% of child-targeted Swiss retail products had a Nutri-Score of D or E, yet favourably graded categories showed notable exceptions. Abreu and Liz Martins described cross-classification mismatches between nutritional quality and degree of processing. Ceccon and Kebbe found that the majority of packaged infant foods in Canada would require a “high-sugar” warning label under stricter nutrient profile models. Critically, Guinot-Barona et al.—including members of the present research group—observed that approximately 68% of processed children’s breakfast products contained high levels of added sugars, a pattern directly aligned with our findings [12,13,14,15,16].

The compensatory design of Nutri-Score—whereby favourable components such as protein, fibre and fruit or vegetable content can offset high sugar levels—is a structural feature common to both the original algorithm and the 2022–2023 update. The limited within-category discrimination documented in this study is therefore attributable to this design characteristic rather than to any specific algorithm version. The 2022–2023 update introduced stricter penalisation of sugars and improved differentiation of sweetened dairy products, which may attenuate—but does not eliminate—the compensation mechanism. The fact that the algorithm version underlying each individual label could not be verified (data collection coincided with the official transition period) is thus a minor source of heterogeneity affecting individual examples, such as the A-rated cereal containing 22.4 g/100 g, rather than the central conclusion, which rests on a design feature shared by both versions [17].

Our findings suggest that caregivers and paediatric dental clinicians should not rely on Nutri-Score grade alone as a proxy for cariogenic risk. Critically reading the ingredients list—paying attention to the position of sugars and the type of sweeteners used—and examining the detailed nutrition facts table remain essential complementary steps. In clinical consultations, paediatric dentists are well positioned to educate families on interpreting nutritional labels beyond front-of-pack grading [9,18].

An alternative front-of-pack approach is the mandatory warning-label system adopted in Chile, Mexico, Peru and other Latin American countries, in which black octagons identify products exceeding predefined thresholds for sugar, sodium, saturated fat or energy. Unlike Nutri-Score, this model is non-compensatory: a product high in sugar is flagged regardless of its other nutritional attributes, a feature directly relevant to the communication of cariogenic risk. Evaluation of the Chilean Law of Food Labelling and Advertising reported a 23.7% reduction in purchases of beverages classified as “high in” after implementation [19]. By contrast, comparative experimental studies conducted across 12 countries found that Nutri-Score outperformed warning symbols in consumers’ objective understanding of overall nutritional quality [20]. These findings suggest that the two systems may have complementary rather than competing roles: graded summary labels may communicate overall nutritional quality more effectively, whereas threshold-based warnings may more reliably identify individual nutrients of concern, particularly sugar. None of the products analysed in the present study displayed a warning label, as no such system is currently mandatory in Spain or across the European Union.

### 4.1. Strengths

Strengths of this study include the systematic evaluation of multiple child-targeted food categories using real commercial products available to consumers in three major retail chains in Valencia, Spain, providing good ecological validity. The simultaneous recording of Nutri-Score grade, total sugar content, and ingredients list composition allowed added sugars to be distinguished qualitatively from intrinsic sugars, an aspect not captured by the Nutri-Score grade or the mandatory nutrition declaration and of direct relevance to cariogenic risk assessment. The explicit interpretation of findings from a paediatric oral health perspective adds a clinically relevant dimension that is underrepresented in existing Nutri-Score research.

### 4.2. Limitations

This study analysed total sugars as reported on mandatory nutrition labels, since current European labelling regulations do not require added sugars to be declared separately. A partial distinction is nonetheless possible through the ingredients list: the presence of terms such as sucrose, glucose syrup, dextrose or honey indicates added sugar, whereas sugar content in products listing only fruit, fruit juice or milk as sugar sources can be attributed to intrinsic sugars. Applying this criterion, products in the juice category contained predominantly intrinsic fructose, while most biscuits, breakfast cereals and desserts contained explicitly added sugars. However, this approach does not allow for quantification of the proportion of total sugar that is added, and intrinsic and added sugars coexist in many formulations (e.g., sweetened dairy products containing both lactose and sucrose); the distinction therefore remains qualitative.

Category-specific analyses should be interpreted as exploratory because of the relatively small number of products included in some food groups. Furthermore, the global Spearman correlation (ρ = 0.515) may partly reflect systematic differences in sugar content and Nutri-Score distribution between food categories rather than a consistent within-category relationship; this limits its interpretation as evidence of algorithm-level discrimination across all food groups.

Although the sample included major child-targeted retail brands available in three large supermarket chains in Valencia, it may not represent the full breadth of the Spanish children’s food market; many infant-specific products do not carry a Nutri-Score label and were thus excluded. Sugar content was obtained from label data without independent chemical verification, and no data on actual consumption patterns or exposure frequency by real children were collected. Several food categories contained groups with very few or single products per Nutri-Score grade (e.g., *n* = 1 for grade A in biscuits and grade C in biscuits), which substantially limits the reliability of within-category statistical comparisons; with such small and unbalanced groups, non-parametric tests may lack power to detect true differences (type II error) while simultaneously producing unstable estimates. Results from these category-level analyses should therefore be treated as purely descriptive and interpreted with extreme caution. The cross-sectional design prevents causal inference. Future studies should incorporate larger samples, chemical analysis distinguishing added from intrinsic sugars, and dietary intake data to estimate population-level cariogenic risk. Additionally, the Nutri-Score grades displayed on products corresponded to labels available at the time of data collection; subsequent product reformulations may have altered the nutritional composition of some items. Additionally, the first phase of data collection was conducted during the official transition period following implementation of the updated Nutri-Score algorithm, and some products collected during that phase may have displayed labels generated using the previous algorithm. Although products collected during the second phase (February–March 2026) would be expected to comply with the updated algorithm, stock turnover and label-version compliance could not be verified. Consequently, some degree of label-version heterogeneity cannot be excluded [8]. This may have introduced label-version heterogeneity across the sample that could not be systematically controlled for.

## 5. Conclusions

Nutri-Score showed a moderate overall association with sugar content; however, its ability to consistently identify child-targeted products with high sugar content varied across food categories. Consequently, Nutri-Score should not be used as the sole indicator of cariogenic potential in child-targeted foods; the nutrition facts panel and ingredients list should be consulted in parallel, particularly when evaluating products intended for children.

## Figures and Tables

**Table 1:** Distribution of products by food category and Nutri-Score grade (*n* = 100).

Category	*n*	A	B	C	D	E	Median Sugar (g/100 g or mL)	Range
**Breakfast cereals**	14	5	3	5	1	0	21.85	1.1–25.0
**Biscuits**	11	1	0	1	5	4	21.00	0.7–45.0
**Breads**	13	0	3	4	4	2	5.70	0.5–13.0
**Milks & milk drinks**	15	1	7	0	3	4	6.90	0.2–61.0
**Dairy products**	12	1	3	4	4	0	7.95	0.5–70.0
**Juices**	7	0	0	3	4	0	5.70	4.1–14.0
**Desserts**	18	0	0	3	6	9	26.50	5.8–59.0
**Frozen foods**	10	0	3	6	1	0	2.10	0.9–9.6
**Total**	100	8	19	26	28	19	—	—

**Table 2:** Sugar content (g/100 g or mL) by Nutri-Score grade across all 100 products.

Nutri-Score	*n*	Median (g/100 g or mL)	Mean (g/100 g or mL)	Min	Max
**A**	8	5.05	6.79	1.1	22.4
**B**	19	4.90	7.11	0.2	22.1
**C**	26	6.60	9.58	0.5	25.0
**D**	28	18.50	19.06	0.5	70.0
**E**	19	32.00	30.49	7.5	61.0

**Table 3:** Summary of inferential statistical results by food category.

Category	*n*	Test	Statistic	*p*-Value	*p* < 0.05 (Uncorrected)	Key Finding
**Breakfast cereals**	14	Kruskal–Wallis	H = 6.70	0.082	No	One A-rated cereal: 22.4 g/100 g
**Biscuits**	11	Kruskal–Wallis	H = 8.72	0.033	Yes *	Clear C → D → E gradient
**Breads**	13	Kruskal–Wallis	H = 1.98	0.576	No	Low sugar, no gradient
**Milks & drinks**	15	Kruskal–Wallis	H = 9.02	0.029	Yes *	High heterogeneity within grades
**Dairy products**	12	Kruskal–Wallis	H = 1.10	0.777	No	Grade D: 0.5–70 g/100 g
**Juices**	7	Mann–Whitney U	U = 7.00	0.857	No	All intrinsic fructose
**Desserts**	18	Kruskal–Wallis	H = 8.31	0.016	Yes *	Sugar increases with grade
**Frozen foods**	10	Kruskal–Wallis	H = 0.79	0.673	No	Low sugar throughout

* *p*-value did not survive Bonferroni correction for multiple comparisons (adjusted α = 0.006 for eight simultaneous tests); results should be considered exploratory.

**Table 4:** Ordinal position of sugar in the ingredients list by Nutri-Score grade (*n* = 100).

Nutri-Score	*n*	Sugar as 1st Ingredient, *n* (%)	Sugar Within Top 3 Ingredients, *n* (%)	Median Sugar Position †
**A**	8	0 (0.0%)	3 (37.5%)	3.0
**B**	19	5 (26.3%)	10 (52.6%)	2.0
**C**	26	6 (23.1%)	16 (61.5%)	3.0
**D**	28	5 (17.9%)	22 (78.6%)	2.0
**E**	19	8 (42.1%)	15 (78.9%)	2.0
**Total**	100	24 (24.0%)	66 (66.0%)	2.0

† Median ordinal rank of the first sugar-related term in the ingredients list, calculated for the 86 products in which a sugar-related ingredient appeared; 14 products had no sugar-related term among the listed ingredients.

## Data Availability

The dataset generated during this study is available from the corresponding author on reasonable request.
